# Modulation of the Tumor-Associated Immuno-Environment by Non-Invasive Physical Plasma

**DOI:** 10.3390/cancers15041073

**Published:** 2023-02-08

**Authors:** Sarah Förster, Yuequn Niu, Benedikt Eggers, Marjan Nokhbehsaim, Franz-Josef Kramer, Sander Bekeschus, Alexander Mustea, Matthias B. Stope

**Affiliations:** 1 Department of Pathology, University Hospital Bonn, 35127 Bonn, Germany; 2 Department of Oral, Maxillofacial and Plastic Surgery, University Hospital Bonn, 53111 Bonn, Germany; 3 Section of Experimental Dento-Maxillo-Facial Medicine, University Hospital Bonn, 53111 Bonn, Germany; 4 ZIK Plasmatis, Leibniz Institute for Plasma Science and Technology (INP), 17489 Greifswald, Germany; 5 Department of Gynecology and Gynecological Oncology, University Hospital Bonn, 53127 Bonn, Germany

**Keywords:** non-invasive physical plasma, cold atmospheric plasma, cold atmospheric pressure plasma, immunology, immune cells, tumor-associated immune response

## Abstract

**Simple Summary:**

Non-invasive physical plasma can be used in various medical applications. As the name suggests, this treatment is non-invasive, as the plasma device is placed over the area to be treated and cold plasma is applied. The main effect of plasma treatment is achieved by reactive oxygen and reactive nitrogen species which induce oxidative stress in the treated sample. Cancer cells are very sensitive to non-invasive physical plasma and plasma treatment induces apoptotic and immunogenic cell death of cancer cells. In the latter, dying cancer cells send out so-called “eat-me” signals which recruit and activate specific immune cells. Non-invasive physical plasma can also directly activate immune cells and increase their aggressiveness (cytotoxicity) towards cancer cells. In addition to cancer and immune cells, plasma treatment affects other parts of the tumor, such as the vasculature, and generally leads to reduced tumor growth.

**Abstract:**

Over the past 15 years, investigating the efficacy of non-invasive physical plasma (NIPP) in cancer treatment as a safe oxidative stress inducer has become an active area of research. So far, most studies focused on the NIPP-induced apoptotic death of tumor cells. However, whether NIPP plays a role in the anti-tumor immune responses need to be deciphered in detail. In this review, we summarized the current knowledge of the potential effects of NIPP on immune cells, tumor–immune interactions, and the immunosuppressive tumor microenvironment. In general, relying on their inherent anti-oxidative defense systems, immune cells show a more resistant character than cancer cells in the NIPP-induced apoptosis, which is an important reason why NIPP is considered promising in cancer management. Moreover, NIPP treatment induces immunogenic cell death of cancer cells, leading to maturation of dendritic cells and activation of cytotoxic CD8^+^ T cells to further eliminate the cancer cells. Some studies also suggest that NIPP treatment may promote anti-tumor immune responses via other mechanisms such as inhibiting tumor angiogenesis and the desmoplasia of tumor stroma. Though more evidence is required, we expect a bright future for applying NIPP in clinical cancer management.

## 1. Introduction

**Figure 1:**
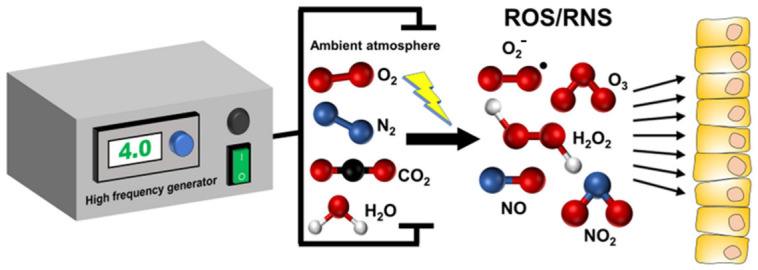
A high-frequency generator transmits energy into the gas between two electrodes, resulting in non-invasive physical plasma (NIPP) formation. The energy transmission to the atoms and molecules of the ambient atmosphere excites these particles and generates charged and radical particles such as reactive oxygen species (ROS) and reactive nitrogen species (RNS). This leads to redox stress in NIPP-treated tissue and induces specific cell responses.

Non-invasive physical plasma (NIPP) is equivalent to a highly reactive and partially ionized gas containing neutral particles (atoms, molecules), charged particles (ions, free electrons), radicals, and electromagnetic radiation. The components of NIPP are in constant interaction with each other, but also with the particles of the ambient atmosphere. This results in energy transmission to the atoms and molecules of the air, producing reactive oxygen species and reactive nitrogen species (ROS/RNS, or RONS), which are primarily responsible for biomedical effects [1] (Figure 1).

In contrast to physical plasma applied in the majority of industrial physical plasma processes at temperatures from a few 100 °C to a few 1000 °C, e.g., for welding and cutting (thermal plasma), NIPP for medical applications has temperatures only slightly above body temperature (non-thermal plasma, cold plasma). Thermal effects can therefore be excluded. The biomedical NIPP effect derives primarily from the signaling effect of ROS and other reactive species [2,3].

In medical applications, NIPP has already been used for many years for tissue regeneration and for treating acute and chronic wounds [4]. NIPP therapies are therefore well established in dermatology and dentistry, with distinct plasma sources generated from different devices, such as the dielectric barrier discharge (DBD) plasma and the gas plasma jet [5,6]. The main therapeutic effects of NIPP treatment include the induction of regenerative processes, as well as a pronounced antimicrobial efficacy [7,8,9]. Here, prolonged treatment time devitalizes degenerated tissue by apoptosis followed by replacing cells from adjacent healthy tissue [10,11]. In this context, NIPP therapy is very tissue-preserving and relatively few post-therapeutic complications occur due to its wound healing and antiseptic properties [12,13].

Since 2005, the number of original studies ranging from cell culture to animal models to investigate the anticancer effects of NIPP has grown exponentially [14]. Among all the potential anti-tumor effects, NIPP-induced apoptosis is most widely studied. Under the oxidative stress produced by plasma, various signaling pathways are involved, such as the activation of MAPK pathways and the suppression of the PI3K/Akt pathway, resulting in cell death [15]. Other potential effects include inducing autophagic cell death [16], pyroptosis [17], ferroptosis [18], regulating cancer cell metabolism [19,20], as well as causing cell cycle arrest, DNA damage, and mitochondrial damage [21].

In oncological NIPP applications, however, immuno-oncological aspects are understudied. The local interaction of the tumor tissue with the immune system is of great importance for the development and progression of cancer [22]. Cancer cells suppress the local immune response and control the activity of tumor-associated immune cells [23,24]. In this review, we highlight pivotal NIPP-induced immunological aspects that might play a role in NIPP application in plasma oncology.

## 2. Effects of NIPP on Immune Cells

A detailed description of the immune system and the complex interplay of immune and cancer cells in the tumor microenvironment (TME) is beyond the scope of this article but can be found in several comprehensive reviews [24,25]. Briefly, the immune system can be divided into innate and adaptive immunity. The former provides immediate, yet unspecific, host defense and is also involved in wound healing and tissue homeostasis, i.e., clearing of cellular debris and dead cells. The latter, consisting of humoral or B cell immunity and cellular or T cell immunity, provides specific antigen-mediated detection and elimination of infectious agents. These specific responses may take days to weeks to develop but include memory functions [22,25]. Despite the separation into innate and adaptive immunity, both arms of the immune system interact closely with each other. Immune functions are carried out by a multitude of leukocytes including cells of myeloid origin such as monocytes, macrophages, and dendritic cells, as well as lymphocytes such as B cells, T cells, and natural killer cells. In the following sections, we summarize the current knowledge on the direct NIPP effects on immune cells. Similar to cancer cell studies on NIPP efficacy, most immunological studies focus on single primary immune cell types or immortalized cell lines, and thus can only represent a small and isolated segment of the immune system. Therefore, where possible, results from co-culture studies or (preclinical) animal models were included in addition to in vitro data.

### 2.1. Peripheral Blood Mononuclear Cells and Lymphocytes

Peripheral blood mononuclear cells (PBMCs) are a mixture of different cell types, mainly various types of lymphocytes and monocytes, and play significant roles in the immune response. Circulating immune cells are quiescent but become activated during recruitment to sites of infection and tissue damage [26].

NIPP treatment induces oxidative stress, which can be indicated by increased intracellular oxidation of 2′,7′-dichlorodihydrofluorescein diacetate (DCF-DA). Increased or prolonged oxidative stress can induce apoptosis and subsequently reduce cell viability. In agreement, NIPP treatment of PBMCs increases oxidative stress levels and leads to a treatment time-dependent induction of apoptosis [27,28]. A comparison of different PBMC subpopulations and their respective cell line equivalents (THP-1 monocytes, Jurkat T lymphocytes) revealed that monocytes are much more robust against NIPP-induced cell death than lymphocytes [29,30,31]. Mitogenic stimulation, i.e., activation of PBMCs by phytohemagglutinin prior to NIPP treatment resulted in increased survival of PBMCs when compared with their quiescent counterparts [27]. Furthermore, PMBCs were shown be more resistant to NIPP-induced cytotoxicity compared to various cancer cell lines [32]. In general, and in agreement with studies in cancer cells, the biomedical effects of NIPP on immune cells were treatment dose- and/or time-dependent.

Only a few studies have focused on the effects of plasma on specific lymphocyte populations. For example, in study focusing on primary CD4^+^ T helper (Th) cells isolated from PBMCs, the authors showed that plasma treatment dose-dependently affected redox signaling (i.e., NIPP increased intracellular glutathione (GSH) concentration) and induced mitochondrial oxidation and depolarization ultimately resulting in apoptosis; furthermore, H_2_O_2_ was shown to be of central importance for NIPP-induced cytotoxicity in lymphocytes [27,28]. A follow-up study analyzed NIPP effects on different CD4^+^ T cell subtypes. T lymphocytes generally can be categorized into naïve cells that have not encountered their antigen yet, and effector/memory cells; these subtypes are characterized by specific marker expression, e.g., CD45RA on naïve vs. CD45RO memory cells, as well as distinct functions in anti-tumor immune responses. Interestingly, NIPP treatment of PBMCs changed the ratio of viable CD4^+^ T cells in favor of naïve cells indicating that memory T cells are more sensitive towards NIPP-mediated redox stress and apoptosis [33]. Similar effects may occur in CD8^+^ T cells, as it has been suggested that cell differentiation state is inversely correlated with resistance to apoptotic cell death as well as anti-tumor capacity [34]. Furthermore, NIPP treatment also drastically decreased the number of CD62L^+^ T cells; CD62L, or L-selectin, is involved in T cell homing to lymphoid tissue. As these and other changes in cell surface marker expression were independent of NF-κB-mediated T cell activation, the authors suggested that rather than directly activating T cells, NIPP favors the survival of so-called terminally differentiated effector memory T cells [33].

Importantly, repeated intraperitoneal injection of NIPP-treated medium did not induce changes in blood leukocyte number and distribution [35], indicating no general activation of lymphocytes or adverse systemic effects of NIPP in mice.

### 2.2. Monocytes/Macrophages

As part of the innate or non-specific immune response, monocytes and macrophages are important effectors and regulators of inflammation. Monocytes circulate in the blood, bone marrow, and spleen and can migrate into tissues upon infection where they can differentiate into macrophages or dendritic cells [26]. In response to different signals such as pathogen- or damage-associated molecular patterns (PAMPs or DAMPs), monocytes/macrophages can generate and release high amounts of ROS and RNS (e.g., O_2_^−^, H_2_O_2_, NO) [36]. In order to withstand the high levels of RONS, monocytes/macrophages have developed several anti-oxidative and cytoprotective mechanisms including increased GSH redox signaling, increased transcription of heme oxygenase 1 (HO1; gene name *HMOX1*), and increased expression of anti-oxidative stress enzymes such as MnSOD or catalase [36]. It has been suggested that these elevated anti-oxidative defense systems render monocytes/macrophages inherently more resistant to ROS-induced cell death [37]. As phagocytic cells, monocytes/macrophages are involved in tissue homeostasis, i.e., the clearance of dead cells and debris as well as pathogen recognition and secretion of inflammatory cytokines [26]. Furthermore, macrophages are important for effective tumor elimination as they serve key roles in recognizing tumor cells and presenting tumor antigens to T and B lymphocytes [38] thereby playing critical roles in both non-specific and specific anti-tumor responses.

The human THP-1 cell line is often used to study monocytes and monocyte-derived cells in vitro [39]; alternatively, primary monocytes can be isolated from PBMCs. NIPP treatment of THP-1 monocytes induced intracellular redox changes, and upregulated *HMOX1* which protects against oxidative stress-induced cytotoxicity and increases the transcription and secretion of pro-inflammatory chemokine interleukin (IL)-8 [40]. As described for other cell types, observed changes increase with prolonged treatment time. Interestingly, NIPP treatment also slightly affected monocyte morphology and the expression of surface markers which are associated with monocyte/macrophage activation, differentiation, or polarization, e.g., HLA-DR and CD163 [40,41]. However, it is currently unclear whether the observed changes reflect monocyte-to-macrophage differentiation. Interestingly, the effects of NIPP on THP-1 surface marker expression were less pronounced than the effects of co-culturing THP-1 monocytes with U87 glioblastoma (GBM) cells; in most cases, additional plasma treatment of either GBM or THP-1 cells did not drastically change surface marker expression or cytokine release in these co-cultures [42]. Nonetheless, plasma-treated THP-1 cells showed enhanced anti-tumor activity towards co-cultured A549 lung carcinoma cells. No cytotoxic effects of control or plasma-treated monocytes were observed when monocytes were co-cultured with non-malignant Beas2B lung epithelial cells [43].

THP-1 monocytes can be differentiated into M0 macrophages by stimulation with phorbol-12-myristate-13-acetate (PMA). M0 macrophages can then be polarized towards pro-inflammatory M1 or anti-inflammatory M2 macrophages characterized by altered surface marker expression and cytokine secretion [24,39]. Kaushik et al. showed that combined treatment of THP-1 monocytes with PMA and NIPP further increased macrophage differentiation when compared to PMA stimulation alone. In fact, based on specific M1/M2-polarization markers such as CD86, CD163, and inducible nitric oxide synthase (iNOS), it was suggested that NIPP may skew macrophage polarization towards M1-like macrophages. This was confirmed by increased cytokine expression and higher cytotoxicity of NIPP-treated macrophages towards co-cultured glioma cells [44]. Similarly, NIPP-treated murine RAW264.7 macrophages decreased the viability of co-cultured GBM or lung carcinoma cell lines [45]. Cytotoxicity seemed to be mediated via increased pro-inflammatory TNFα release [44,45]. Altered surface marker expression after NIPP treatment has also been shown for CD11b^+^ F4/80^+^ murine bone marrow-derived macrophages [46]. Furthermore, NIPP treatment of macrophages has been shown to induce macrophage migration and invasion [38,45].

### 2.3. Dendritic Cells

Like macrophages (and B cells), dendritic cells (DCs) belong to the family of professional antigen-presenting cells and are important players in both innate and adaptive immune responses. The main functions of DCs include the phagocytosis and processing of exogenous antigens (including tumor-associated antigens) and, after migration into secondary lymphoid organs, antigen presentation and T cell activation. By releasing cytokines, DCs are also involved in inflammatory processes [47,48].

DCs, like monocytes and macrophages, are highly resistant to oxidative stress, and ROS are involved in DC maturation and functions [49,50]. To study the effects of NIPP on DCs, DCs can be differentiated from human monocytes as previously described [32,51,52]. These monocyte-derived DCs (moDCs) are characterized by the expression of specific cell surface markers such as CD11c, and receptors essential for antigen presentation, e.g., HLA-DR as well as co-stimulatory molecules such as CD80 and CD86 [47,52]. In line with previous reports, moDCs showed time-dependent reduced metabolic activity and viability after NIPP treatment, but were markedly more resistant to NIPP-induced cytotoxicity than lymphocytes. Furthermore, while indirect treatment with NIPP-treated phosphate-buffered saline (pPBS) had no significant effects on moDCs, both direct treatment with NIPP and co-culture with NIPP-treated cancer cells slightly induced DC-specific surface marker expression (e.g., CD86) and resulted in a mild increase in pro-inflammatory cytokine release (e.g., IL-6, TNFα) [51,52]. Similar results were observed in co-cultures of murine bone marrow-derived DCs and plasma-treated murine cancer cells [53]. Exposure of moDCs to lysates of NIPP-treated A375 melanoma cells also augmented the maturation potential, as shown by increased marker expression and cytokine secretion [32]. In addition, several groups could show that NIPP treatment of glioma, pancreatic, and colon cancer cells resulted in more efficient phagocytosis of these cells by DCs when compared to untreated control cells [51,54,55]; the authors suggested that this effect may be due to increased expression of “eat-me” signals and the induction of immunogenic cell death after NIPP treatment (discussed in more detail in Section 3.1) [51,55]. So, while direct NIPP treatment may not be sufficient to induce full DC differentiation and activation, NIPP may still improve DC’s anti-tumor activity. In agreement, NIPP has been shown to affect the interplay of DCs with T cells and to induce specific T cell polarization. In co-culture studies with allogeneic T cells, mature moDCs loaded with NIPP-treated cancer cell lysates induced cytotoxic CD8^+^ T cells and Th17 cells while preserving Th1 response, and did not induce pro-tumorigenic Th2 and regulatory T cells (Tregs). These results indicate that NIPP may stimulate favorable anti-tumor functions of DCs. Preliminary evidence also suggests that priming of T cells with DCs exposed to NIPP-treated A375 melanoma cell lysates results in higher cytotoxic activity of CD8^+^ T cells against live A375 cells compared to untreated controls [32]. In vivo studies confirmed the potential anticancer effects of NIPP-treated DCs. Mice receiving adjuvant NIPP treatment after surgical resection of orthotopic breast cancer tumors showed an increased percentage of mature CD86^+^ and CD80^+^ DCs in tumor-draining lymph nodes, and increased numbers of tumor-infiltrating CD4^+^ and CD8^+^ T cells. Anti-tumor efficacy of NIPP was further validated by reduced tumor regrowth and prolonged survival of mice receiving adjuvant NIPP treatment compared to the untreated (sham surgery) group [53]. Similar results were reported in melanoma-bearing mice receiving transdermal NIPP treatment [56].

### 2.4. Other Immune Cells

Currently, only one report investigated the effects of NIPP treatment on neutrophils and natural killer (NK) cells. As described for macrophages and DCs, NK cells show enhanced cytotoxicity towards NIPP-treated skin cancer cells. Co-culture of plasma-treated cells with untreated NK cells also induced changes in pro-inflammatory cytokine secretion, e.g., increased IL-8. These effects were not observed when NK cells were co-cultured with NIPP-treated non-malignant HaCaT keratinocytes [57]. As described for other cells, NIPP treatment also reduced neutrophil metabolic activity. Interestingly, while NIPP did not affect the bactericidal activity of neutrophils, it did increase superoxide release and neutrophil extracellular trap (NET) formation [58]. The potential relevance of these findings in the context of cancer remains to be investigated.

## 3. Effects of NIPP on Tumor–Immune Interaction

In the process of anti-tumor immune response, there are two crucial processes: (i) DCs present the cancer antigens to CD8^+^ T cells (priming), and (ii) primed CD8^+^ T cells recognize and kill the tumor cells. However, the cancer cells may escape the two steps by various mechanisms [59]. The immunosuppressive properties of TME can suppress the immunogenic elimination of tumor cells, and thus prevent immunotherapy [60]. During cancer cell treatment, NIPP also modulates the expression of various matrix effectors such as matrix metalloproteases and inflammatory mediators, which strongly suggests a complex interaction of NIPP-treated tumor cells with the TME [61]. In addition to directly inhibiting cancer cell survival, evidence has shown that NIPP treatment also targets the tumor–immune interaction, thereby attenuating the cancer cell immune evasion. Moreover, the effects of NIPP on other parts of the TME such as fibroblasts, extracellular matrix (ECM), and endothelial cells have also been investigated [62]. Therefore, it is of great significance to further discuss how NIPP treatment affects the tumor–immune interaction and alters the immunosuppressive properties of the TME.

### 3.1. NIPP Induces Immunogenic Cell Death

To develop strategies targeting tumor immune evasion, plenty of studies focus on the emerging concept of immunogenic cell death (ICD). When cancer cells undergo homeostatic programmed cell death (PCD), which often occurs in the form of apoptosis, the dying cells are considered tolerogenic or non-immunogenic, i.e., barely impacting the immune system. In contrast, ICD in cancer is a cell death modality in which the dying cancer cells act as a vaccine to trigger the immune system and elicit specific anti-tumor immune responses. ICD characteristics include surface-exposed calreticulin (CRT) on the plasma membrane, increased secretion of adenosine triphosphate (ATP), and release of high-mobility group box 1 (HMGB1) protein. These enhanced “eat-me” signals further activate the immune responses of DCs and T cells [63,64].

Like chemotherapy, radiotherapy, and photodynamic therapy, NIPP treatment has also been able to sensitize the TME and induce ICD of cancer cells by regulating the cellular redox homeostasis [65,66]. The first report regarding NIPP-induced ICD in cancer dates back to 2015. After NIPP exposure, nasopharyngeal carcinoma CNE-1 cells revealed promoted secretion of ATP and increased expression of endoplasmic reticulum stress proteins ATF4 and STC2, which might further augment the anti-tumor effects of the M0 THP-1 macrophages [67]. Similarly, NIPP treatment enhanced ATP secretion and surface-exposed CRT in A549 lung cancer cells, which could be reversed by the treatment with antioxidants such as N-acetyl cysteine, indicating the key role of the oxidative stress pathways in such NIPP-induced ICD [68]. These in vitro data shed light on the potential of NIPP-induced ICD in cancer treatment. However, the multidimensional processes involved in ICD cannot be studied in in vitro model systems [63]. The first in vivo evidence of NIPP-induced ICD was provided by Lin et al. in a syngeneic CT26 colorectal carcinoma model in Balb/c mice. CT26 cells were treated with NIPP (ICD-inducer) or cisplatin (non-ICD inducer) and untreated media as control in vitro. The mice were then immunized with these treated CT26 cells one week before being re-challenged by live CT26 cells. Results showed that immunization with NIPP-treated cancer cells significantly inhibited tumor growth [69]. Such tumor cell vaccination assay is essential for validating in vivo ICD [63]. The authors also validated the direct ICD-inducing effect of NIPP treatment in vivo. The Balb/c mice with subcutaneous CT26 colorectal tumors were exposed to NIPP treatment, which promoted the expression of CRT and HMGB1. The recruitment of immune cells including CD45^+^ leukocytes and CD11c^+^ DCs were also enhanced [69].

As the skin is the most accessible organ of the human body, which is convenient for the operation of NIPP therapy, dermatology is one of the major application scenarios of NIPP [70]. Therefore, melanoma has an advantage in the research of NIPP application in cancer, particularly for the in vivo models. In a melanoma mouse model, Lin and colleagues showed that the direct treatment of tumors with NIPP suppressed tumor growth and prolonged survival. Moreover, a transient but significant increase in CRT expression was observed, while the expression of the anti-phagocytic signal CD47 remained unchanged. The antigen presentation by DCs and the cytotoxic immune responses by T cells were also enhanced, indicating the stimulation of systemic anticancer immunity [71]. Similarly, Bekeschus et al. tested different NIPP device setups and found that He/O_2_-generated NIPP significantly reduced tumor burden in the B16F10 melanoma mouse model, where elevated intratumor levels of CD8^+^ T cells and DCs were observed, too. In addition, NIPP-induced ICD was further validated by the tumor cell vaccination trial [72]. These studies highlight the potential of NIPP serving as an ICD-inducer in anticancer therapy.

Although plenty of studies have shown consistent results that NIPP treatment can induce ICD in cancer, the molecular mechanism remains unclear. It has been reported that the combination of pulsed electric fields and PBS containing persistent RONS (including H_2_O_2_, NO_2_^−^, NO_3_^−^) could not elevate the expression of surface-exposed CRT to a comparable level of NIPP treatment in B16F10 and A375 melanoma cells. Thus, the NIPP-generated short-lived (lifetimes <1 s) RONS such as hydroxyl radicals, atomic oxygen, and nitric oxide were considered the main effectors of ICD [73]. However, whether the solutions pretreated by NIPP could act as ICD-inducers in cancer is controversial. It has been shown that NIPP-treated liquid media rich in H_2_O_2_ could suppress tumor cell proliferation, increase CRT exposure, and elevate ATP release in melanoma cells Hmel1 and HBL, and pancreatic cancer cells PANC-1 [74]. Another study also reported that in pancreatic cancer cell lines, particularly MIA-Paca-2 and PANC-1, pPBS could induce ICD signals including surface-presented CRT, secretion of ATP, HMGB1 release, and downregulation of CD47, which further promoted maturation of and phagocytosis by DCs. Interestingly, non-immunogenic cell death was observed in the immunosuppressive pancreatic stellate cells (PSCs) after pPBS treatment, suggesting there might be specific mechanisms of NIPP-induced ICD in cancer cells [51]. Similarly, upregulation of ICD markers after in vitro treatment of pPBS was also validated in PDA6606 pancreatic cancer cells as well as colon cancer cell lines CT26 and MC38. ICD was not induced in non-malignant HaCaT keratinocytes [75]. Of note, in addition to these reported in vitro applications, repetitive treatment of pPBS also significantly reduced tumor mass in a CT26 peritoneal carcinomatosis mouse model [75]. Recently, a study by Miebach et al. also validated the anticancer effects of the NIPP-treated saline in the syngeneic peritoneal carcinomatosis mouse model. Intriguingly, they also showed equal efficacy of NIPP-oxidized medical-grade sodium chloride (oxNaCl) and control NaCl solution supplemented with concentration-matched H_2_O_2_ and NO_3_^−^ (cmc) in inducing cytotoxicity, increasing tumor cell immunogenicity, and activating anti-tumor immune responses [76].

In addition, there could be synergistic effects when combining pPBS and agents which suppress the function of the endogenous antioxidant system. For instance, the combination treatment of pPBS and the thioredoxin reductase 1 inhibitor auranofin induced apoptosis and ferroptosis in GBM cell lines, concomitant with enhanced ICD signals and DC maturation. In vivo application of this combination inhibited tumor growth and improved survival, suggesting a potential therapeutic strategy of combining NIPP with other synergistic agents [55]. Another promising example is the combined use of plasma treatment with immune checkpoint inhibitor (ICI) to further augment the anti-tumor immune responses. Chen et al. described a NIPP application to a B16F10 melanoma mouse model, which facilitated the delivery of NIPP through the skin into tumor tissue, resulting in ICD, maturation of DCs, and activation of T cells; moreover, the combined use of NIPP with anti-programmed death-ligand 1 antibodies further enhanced the T cell anti-tumor immune responses. This combination of NIPP with ICI therapy showed better efficacy than either treatment alone [56]. So far, research provides robust evidence to support NIPP acting as a promising ICD inducer in anticancer therapy. It is also imperative to further investigate the underlying mechanisms and explore the potential application of NIPP in clinical cancer management.

### 3.2. NIPP Ameliorates the Immunosuppressive TME

The abnormal angiogenesis and the concurrent hypoxic surroundings account for an important part of the immunosuppressive TME. Such malfunction of the tumor perfusion leads to the preferential recruitment and activation of immunosuppressive tumor-associated macrophages (TAMs) and Tregs in TME, which prevents the effector lymphocytes from reaching and killing the tumor cells [77]. In addition, desmoplasia in TME induced by poor tissue oxygenation and chronic inflammation also blocks the movement of the anti-tumor immune cells towards the tumor lesions [62]. Therefore, it is significant to investigate the role of NIPP in tumor vascularization and ECM remodeling.

In wound healing, NIPP can promote angiogenesis and tissue regeneration, inducing key factors such as vascular endothelial growth factor (VEGF), hypoxia-inducible factor 1-alpha, fibronectin, and collagen [78,79,80]. However, NIPP appears to inhibit angiogenesis in tumor tissue.

It has been reported that NIPP treatment decreased the level of VEGF in various malignancies, such as breast cancer [81], melanoma [82], and osteosarcoma [83]. Furthermore, NIPP treatment caused metabolism reduction, apoptosis induction, and inhibited migration and tube formation in the human endothelial cell line HDMEC [84]. Using a common method for angiogenic study, the chorioallantoic membrane assay, Kugler and colleagues showed that NIPP treatment significantly inhibited angiogenesis of HuH-7 hepatoma xenografts in chicken egg as significantly reduced intratumoral vessel density was observed. In addition, via intravital fluorescence microscopy, they also found that NIPP treatment increased vascular permeability and caused occlusions in the tumor-associated blood vessels. However, the downregulation of VEGF was not statistically significant. Therefore, the authors attributed anti-angiogenic effects of NIPP to the direct effects on blood vessels, such as coagulation [85]. Of note, it has been reported that NIPP induces blood clotting through a nonthermal mechanism, while platelet activation by ROS, particularly H_2_O_2_, was considered as the central event in NIPP-induced coagulation [86]. Consistently, this inhibitory role of NIPP in angiogenesis was also validated through the aortic ring assay, in which 60 s exposure to NIPP significantly decreased the sprouting distance of newly formed vessels of excised rat thoracic aortic segments [87]. However, a study employing squamous cell carcinoma and melanoma mouse models reported different results. Although NIPP treatment significantly suppressed tumor growth, the number of CD31-positive vessels was not affected, indicating that in vivo tumor angiogenesis was not regulated by NIPP treatment [88]. Therefore, the role of NIPP in tumor angiogenesis remains unclear. Furthermore, it must be considered that NIPP treatment is highly dose- and device-dependent [89]. L929 mouse fibroblasts showed an increase in cell viability and collagen expression after 15 s NIPP treatment, whereas a 25 s treatment induced a significant inhibition [90].

Cancer-associated fibroblasts (CAFs) play a central role in the overproduction of collagen and the stiffness of ECM in TME, which leads to impaired anti-tumor immune responses and therapy resistance [91]. Analogous to angiogenesis, NIPP treatment is also thought to induce fibroplasia and fibrillogenesis in wound healing [92]. However, NIPP’s functionality in tumor ECM remodeling remains to be further elucidated. In addition to the aforementioned treatment time-dependent role, the reported effects of NIPP on fibroblasts also varied by research model. For example, it has been shown that NIPP exposure led to senescence of primary human skin fibroblasts, which were seeded in six-well plates when treated [93]. At the same time, no effect on dermal ECM production and degradation was observed when NIPP was applied to a 3D human dermal substitute generated from primary human dermal fibroblasts, though such treatment did induce apoptosis and inhibited growth in the HCT-116 3D tumor spheroid model [94]. Despite the controversy over the effects of NIPP treatment on normal fibroblasts, promising data have been reported in the scenario of pathological conditions. For example, using an experimental murine model of scleroderma in which dermal fibrosis was induced by bleomycin, Arndt et al. found that NIPP-treated mice manifested significantly reduced dermal thickness and collagen deposition [95]. Another study compared the NIPP effects on normal fibroblast (NFs) and keloid fibroblasts (KFs). Interestingly, NIPP promoted collagen production and cell migration in NFs, which were inhibited in NIPP-treated KFs [96]. Of note, CAFs and KFs are functionally similar in overproducing collagen [62]; meanwhile, the CAFs in TME not only restrict anti-tumor immune cells by rigidifying the ECM, but also directly interact with them via the secretion of various effector molecules, leading to an immunosuppressive TME [97]. Despite such important roles of CAFs in TME, current knowledge of the involvement of NIPP in CAF regulation is still very scarce.

Briefly, in contrast to its role in wound healing, NIPP treatment in cancer tends to inhibit angiogenesis and desmoplasia, thereby reshaping TME, mitigating immune evasion, and preventing tumor progression. Due to conflicting results, the data are not yet sufficient. Nevertheless, it appears promising that NIPP can be used to ameliorate immunosuppressive TME.

## 4. Clinical Applications of NIPP in Cancer Therapy

In previous clinical trials which focused on wound care and ulcer management, NIPP treatment has been proven to be safe, painless, and well-tolerated, with few NIPP-related adverse events and no serious adverse events reported [98,99,100]. Despite the low levels of radiation exposure in NIPP treatment, a five-year follow-up study investigating patients who received NIPP treatment for wound healing showed no malignant or pathological changes, which further validated the safety of the clinical application of NIPP [101]. Moreover, the feasibility of using NIPP as at least a part of a clinical cancer therapy has also been studied. Several studies demonstrated the safety and clinical efficacy of NIPP treatment in advanced head and neck squamous cell carcinoma (HNSCC) and strongly suggested potential benefits for cancer patients’ care [102,103]. In one recent clinical application, six patients with inoperable HNSCC with open infected ulcerations were treated with NIPP. Treatment consisted of cycles of three single applications (1 min/cm^2^) per week, followed by a week with no treatment. In addition to reduced pain and odor medication requirement and palliative benefits in terms of quality of life, NIPP treatment resulted in partial, albeit not sustainable, remission in two patients. Biopsy at remission revealed moderate numbers of apoptotic tumor cells and a desmoplastic reaction of the connective tissue with increased production of ECM. Notably, NIPP-treated tumor tissue was almost devoid of CD11b^+^ myeloid cells. Whether this deficiency was a direct effect of NIPP treatment or an indirect effect of reduced infectious load remains unclear [13]. In addition, NIPP can also be used as an assistant therapy in cancer management, such as reducing the side effects of cancer therapy. Recently, it has been reported that NIPP is safe and feasible in managing radiation dermatitis of breast cancer patients, which is the most common acute side effect caused by breast cancer radiotherapy [104]. Nonetheless, research on the application of NIPP in clinical cancer therapy is still in its infancy. Currently, only a few clinical trials are registered for employing NIPP to treat malignant or precancerous lesions, highlighting the significance of multi-centered, large-scale studies in the future [105].

## 5. Conclusions and Future Perspectives

**Figure 2:**
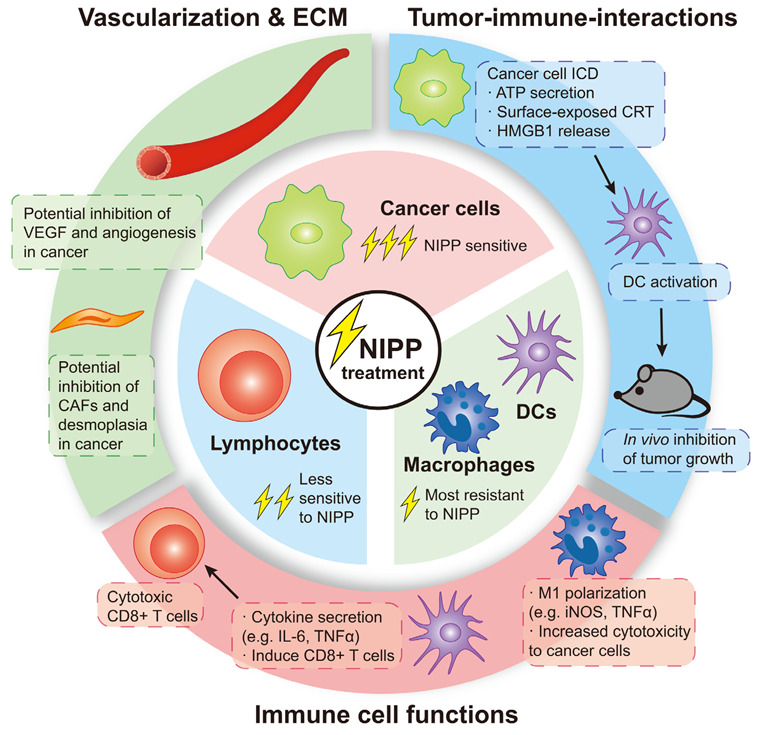
NIPP treatment induces a plethora of reactions in cancer cells and the tumor microenvironment (TME). NIPP kills tumor cells in vitro and inhibits tumor growth in vivo. This can be partly explained by the fact that cancer cells are generally more sensitive towards NIPP-induced apoptosis when compared to immune cells. Importantly, NIPP induces immunogenic cell death (ICD) of cancer cells which activates and recruits immune cells such as DCs and cytotoxic T cells. The molecular mechanism remains to be fully elucidated, but in vitro studies on immune cells corroborate these findings. For example, NIPP treatment of monocytes/macrophages and DCs induces pro-inflammatory cytokine secretion and increases cytotoxicity towards co-cultured cancer cells. DCs loaded with NIPP-treated cancer cells are more effective in priming CD8^+^ cells, further increasing their cytotoxicity. NIPP also affects other parts of the TME including endothelial cells and cancer-associated fibroblasts. The role of NIPP in tumor angiogenesis and ECM remodeling is currently not well understood but may reveal similar synergisms for cancer treatment as shown for cancer and immune cells.

NIPP efficacy on cancer cells has been studied extensively. In this review, current knowledge of the role of NIPP in regulating TME and immune cells including the communication between immune and cancer cells, represents NIPP as a promising tool for preventing tumor cells from immune evasion. In general, even if direct NIPP effects on immune cells alone are not as strong or clear as hoped, co-culture studies and xenografts point towards synergistic benefits with no adverse effect and no disadvantage for additional NIPP treatment in cancer therapy (Table 1, Figure 2). However, there are still challenges for future investigation. Firstly, studies so far have reported heterogenous NIPP treatment regimens, using different NIPP devices, different treatment times, and different time points after treatment for various analyses, which impede the replication of results and the standardization of application. For example, effects of DBD plasma can be influenced by air humidity and temperature [106]. It has also been shown that the RONS species in the cell culture media induced by DBD plasma could be cell type-dependent [31]. For plasma jet, the standardization is also a critical issue, especially when operating manually, which highlights the importance of using reproducible plasma treatment controlled by a computer program [27]. Furthermore, only a few studies took measures to compensate the secondary effects due to the gas flow of plasma jet, such as liquid evaporation, molarity change, and pH level alteration [29,30,35]. Such heterogeneity brings difficulties for the comparison and interpretation across different studies, calling for reliable and standardized protocols [107]. Secondly, NIPP treatment induces a combination of various components and reactions, towards which the understanding to date is limited to the general induction of oxidative stress; whether there are key substances that play major roles and the corresponding mechanisms of such are still unclear. In addition, due to the convenience of performance, current clinical application of NIPP in cancer treatment mainly focuses on neoplasms and their complications on the skin. In fact, there are obstacles of NIPP treatment even for skin applications as well. For instance, it is difficult for the DBD plasma to ignite uniformly on the irregular surfaces. To solve such problem, flexible DBD devices might be valuable for future investigation [108,109]. Another essential topic is the side effects of NIPP treatment. Tolerable side effects such as bleeding and erythema have been recently reported in the clinical applications of plasma jet on HNSCC patients, which are attributed to stroma reactions [102,103]. However, detailed interpretation such as whether those side effects are associated with desiccation or local hypoxia caused by plasma jet is still missing. Furthermore, in addition to employing NIPP in the treatment of tumors on the body’s surface, progression has also been achieved to make NIPP endoscopically usable to treat the tumors which are harder to access, such as cholangiocarcinoma [110] and glioblastoma [111]. Last but not least, considering the enhancing effects of NIPP on immune cells, it is also promising to synergistically use NIPP combined with immunotherapy in the future, such as boosting immune sensitivity and reducing side effects in the chimeric antigen receptors (CAR)-T cell therapy [112]. In summary, NIPP plays an important role in modulating the tumor-associated immuno-environment. As a safe and economical therapeutical procedure, NIPP technology is developing rapidly for its application in cancer management. Wider use in clinical practice could be expected in the future.

## Figures and Tables

**Table 1:** Representative study results using NIPP in the context of tumor–immune interaction.

Plasma Source	Cell Type/Model System	Study Type	Treatment	Controls	No. of Replications	Main NIPP Effects
Plasma jet	PBMCs	In vitro (primary cells)	1× 5, 20, 60 s	Untreated (0 s) and Argon gas control	*n* = 4–12 12 donors	Monocytes are more resistant to oxidation and cell death than lymphocytes [27]
DBD	Monocytes/Macrophages (THP-1 cells) and T cells (Jurkat cells)	In vitro (co-culture)	1× 30, 45, 60, 75, 90, 105 Hz	Untreated (0 Hz)	*n* ≥ 3	Increase of DAMPs on NIPP-treated cells stimulates migration of and phagocytosis by monocytes and macrophages [31]
Plasma jet	Tumor model (6606PDA pancreatic cancer cells in C57BL/6 mice)	In vivo (mouse model)	Daily i.p. injection (indirect ^+^, 10 min) for up to 35 days	Untreated medium	6–25 mice per group	Repeated treatment prolongs survival by reducing tumor burden and inducing tumor cell apoptosis and has no systemic side effects [35]
Plasma jet	Monocytes/Macrophages (THP-1 cells and cells isolated from PBMCs)	In vitro (cell lines and primary cells)	1× 10, 20, 120 s	Untreated (0 s)	*n* = 3–8 3 donors	Altered cell surface marker expression and cytokine secretion in monocytes [41]
Plasma jet and DBD	Patient-derived GBM samples	Ex vivo (GBM tissue biopsies)	1× 30, 120 s	Untreated (0 s)	16 patient biopsies	Induction of apoptosis in patient-derived GBM samples and altered cytokine, chemokine, and growth factor release ex vivo [42]
DBD	Monocytes/Macrophages (THP-1 cells) and Cancer or normal cells (A549 lung carcinoma or Beas2B epithelial cell)	In vitro (co-culture)	50, 100, 300, 700 mJ	Untreated (0 mJ)	*n* ≥ 2	Immune cells and non-cancerous cells are more resistant to NIPP than cancer cells; NIPP-treated macrophages show increased anti-tumor activity in vitro [43]
Plasma jet	moDCs (from PBMCs) and Cancer or stellate cells (pancreatic MIA-Paca-2, PANC-1, BxPC3, Capan-2; hPSC21, hPSC128, RLT-PSC cells)	In vitro (co-culture)	1× 5 min (indirect ^+^)	Untreated PBS	*n* = 3 4 donors	Dose-dependent induction of ICD and improved phagocytosis of NIPP-treated cells by DCs [51]
Plasma jet	moDCs (from PBMCs)	In vitro (primary cells)	1× 60, 120, 180 s	Untreated (0 s) and Argon gas control	*n* = 3–6 6 donors	Dose-dependent decrease of metabolic activity and viability of DCs as well as increases DC marker gene expression and cytokine release [52]
Plasma jet	Postsurgical tumor model (4T1 breast cancer or B16F10 melanoma cells in BALB/c or C57BL/6 mice)	In vivo (mouse model)	1× 1, 2, 3, 4 min	Untreated (0 s)	6–7 mice/group	Reduction of tumor progression accompanied by increase of mature DCs in tumor-draining lymph nodes as well as intratumoral T cells [53]
DBD	Cancer cells (CNE-1 nasopharyngeal carcinoma cells)	In vitro (cell lines)	1× 47, 141, 282, 705 mJ	Untreated (0 mJ)	*n* = 3	Increased ATP secretion, exposed-CRT, and expression of ER stress proteins [67]
DBD	Cancer cells (CT26 colorectal carcinoma cells; murine) Syngeneic tumor model (CT26 cells in Balb/c mice)	In vitro (tumor cell vaccination assay) In vivo (mouse model)	In vitro: 1× 10 s/29 kV/30 Hz In vivo: 1× daily for 5 days, 10, 25, 50 s/29 kV/750 Hz	In vitro: cisplatin or media only; In vivo: untreated mice	*n* = 10	Immunization with NIPP-treated cancer cells inhibits tumor growth; In vivo NIPP treatment enhances CRT expression and immune cell recruitment [69]
DBD	Subcutaneous tumor model (B16F10 melanoma cells in C57BL/6J mice)	In vivo (mouse model)	Daily for 5 days; 30 kV, 700 Hz	Untreated mice	8–11 mice per group	Direct NIPP treatment prolongs mice survival, increases CRT expression, and enhances the antigen presentation by DCs and the cytotoxic immune responses by T cells [71]
Plasma jet	NIPP-activated saline; Syngeneic peritoneal carcinomatosis model by i.p. injection of CT26 cells in Balb/c mice	In vitro generation of NIPP-activated saline; in vivo application (mouse model)	Indirect ^+^, i.p. injection in every 2 days, 5 injections in total	Normal NaCl saline treatment	*n* = 8	NIPP-activated saline suppresses tumor growth and increases tumor cell immunogenicity [76]
Surface micro discharge (SMD)	Chorioallantoic membrane (CAM) assay (HuH7 hepatocellular carcinoma cells in chicken embryos)	In vivo (CAM assay)	60 s per day for 4 days	Sham treatment	*n* = 17–51	Decreased intratumoral vessel density [85]

^+^ Indirect treatment indicates treatment of cells with plasma-treated solutions as opposed to direct treatment of cells in medium.

## Data Availability

All cited publications have been published.
